# The relevance of a rodent cohort in the Consortium on Individual Development

**DOI:** 10.1016/j.dcn.2020.100846

**Published:** 2020-08-27

**Authors:** Rixt van der Veen, Valeria Bonapersona, Marian Joëls

**Affiliations:** a Dept. Translational Neuroscience, UMC Utrecht Brain Center, University Medical Center Utrecht, Utrecht University, Utrecht, the Netherlands; b Faculty of Social and Behavioral Sciences, Leiden University, Leiden, the Netherlands; c University of Groningen, University Medical Center Groningen, Groningen, the Netherlands

**Keywords:** Rodent models, Early life stress, Maternal care, Social behavior, Translational, Systematic literature search

## Abstract

One of the features of the Consortium on Individual Development is the existence of a rodent cohort, in parallel with the human cohorts. Here we give an overview of the current status. We first elaborate on the choice of rat and mouse models mimicking early life adverse or beneficial conditions during development. We performed a systematic literature search on early life adversity and adult social behavior to address the status quo. Next, we describe the behavioral tasks we used and designed to examine behavioral control and social competence in rodents. The results so far indicate that manipulation of the environment in the first postnatal week only subtly affects social behavior. Stronger effects were seen in the model that targeted early adolescence; once adult, these rats are characterized by increased attention, a higher degree of impulsiveness and reduced social interest in peers. Many experiments in our rodent models with tightly controlled conditions were inspired by findings in human cohorts, and now allow in-depth mechanistic investigations. Vice versa, some of the findings in rodents are currently followed up by dedicated investigations in the human cohorts. This exemplifies the added value of animal investigations in a consortium encompassing primarily human developmental cohorts.

## 1 Introduction

Environmental conditions, particularly those experienced during early development, extensively influence the structure and functional connectivity of the human and rodent brain (Glynn and Baram, 2019; Miguel et al., 2019). This has been frequently demonstrated for children experiencing adverse conditions during the perinatal period, early childhood or adolescence (Teicher et al., 2016). Endocrine regulatory pathways have been postulated to be important in the life history theory and specifically cortisol and the HPA-axis are thought to mediate early life effects on later life development (Worthman and Kuzara, 2005). It is therefore not surprising that early life adversity (ELA) also affects behavioral performance, that directly depends on brain structure and functional connectivity, as well as hormonal (dis)balance. For instance, many studies point to a higher risk on impaired mnemonic function, aberrant reward-based decision-making and enhanced anxiety-related behavior after early life adversity (Delavari et al., 2016; Herzberg and Gunnar, 2019; Woldemichael et al., 2014). Conversely, a favorable rearing environment might have beneficial effects on cognition, emotionality and response to stress (Cirulli et al., 2010; Morè et al., 2019).

The changes in behavioral performance related to early life adversity add to the risk to develop psychopathology, especially in those individuals that are genetically predisposed (Heim and Binder, 2012). The causative relation between early life environment and later life behavioral performance, however, is hard to establish in humans, partly due to the lack of (experimental) control over the environment, resulting in many factors that need to be taken into account when interpreting the results. Even in a prospective design -lacking the recall bias of a retrospective cross-sectional approach-, factors hard to control for and as diverse as socioeconomic status, eating habits or peer pressure strongly influence the link between early life environment and cognitive performance later on. This link can be studied in a much more straightforward manner in rodent cohorts, with precise control over many environmental factors, like housing conditions, access to food, temperature, litter size or exposure to conspecifics (Knop et al., 2017). Moreover, animal studies allow for good control over the genetic background of subjects e.g. by reducing (or enhancing) the expression of particular genes by design. Invasive approaches in animals can furthermore contribute to delineating pathways and mechanisms underlying the changes evoked by early life conditions. Finally, generations in rodents encompass months instead of decades, as in human cohorts. This facilitates the investigation of heritable traits (see e.g. Clinton et al., 2014; Stead et al., 2006) and cross-generational effects in the face of varying rearing environments (Langenhof and Komdeur, 2018; Van Cann et al., 2019; van Steenwyk et al., 2018). For all of these reasons, the Consortium on Individual Development (CID) decided to run a rodent cohort in parallel with the many human cohorts. The rodent cohort aims to study how early life conditions change brain function and behavior (link with human work that focuses on changes in the human brain (potentially) contributing to the development of social competence and behavioral control); how this can be readjusted by interventions (link with human work that studies to what extent children at various stages of development benefit from familial intervention programs, in terms of their social competence and behavioral control); and whether or not effects persist into the second generation (link with human work studying the influence of gene x environment interactions in parents on the development of social competence and behavioral control in their offspring). To promote the translation of findings in the rodent cohort to the human cohorts, the rodent studies included (but were not restricted to) the same behavioral endpoints as in humans, i.e. behavioral control and social competence, reflecting indices for those who thrive in life and who do not.

**Fig. 1:**
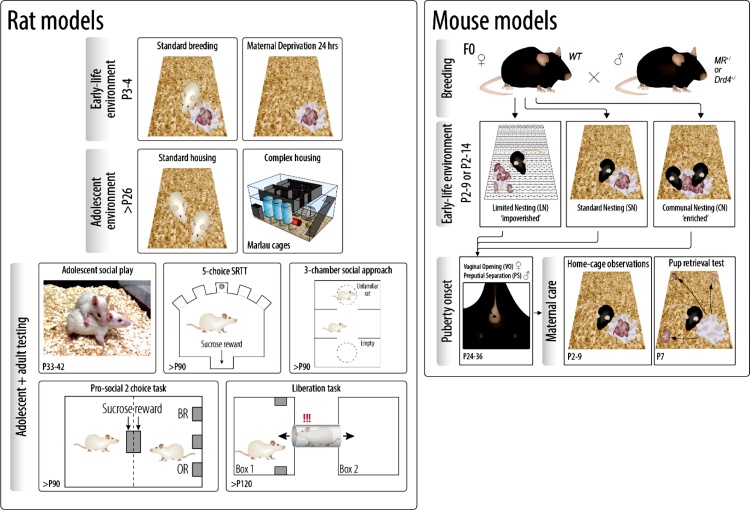
Study designs for our rat and mouse models. *Rat models:* Litters were either standard bred or exposed to maternal deprivation for 24 h on postnatal day 3-4. In adolescence, rats were housed in standard cages (standard housing) or in Marlau cages (complex housing). In adolescence social play was measured and in adulthood different batches of animals were tested in the 5-choice serial reaction time task (5-choice SRTT), 3-chamber social approach task, prosocial 2- choice task or the liberation task. *Mouse models:* A wild-type female mouse was paired with a heterozygous MR^+/−^ or DRD4^+/−^ male to obtain mixed litters. Litters were put in the early environmental conditions from postnatal day 2 to 9 in the MR^+/−^ stduy and from day 2 to 14 in the DRD4^+/−^ study. Puberty onset was measured in adolescence and maternal care of the F1 generation was observed in adulthood. P = postnatal. Figure design by Jelle Knop, partly based on (Knop et al., 2020, [Knop et al., 2019] 2019).

In this paper, we first describe several rearing conditions that were used in the animal cohort. The first set of models (Section 2.1) taps on the mother- offspring interaction being one of the most important environmental determinants early in life. Some models that we used are characterized by impaired maternal care received by the pups, e.g. by taking the mother away for 24 h or exposing the dam (and her litter) to impoverished housing conditions. Importantly we not only studied the negative part of the spectrum but also included the condition of communal nesting, which is thought to improve the quality of maternal care received by the pups. This also creates the opportunity to test differential susceptibility, i.e. the phenomenon that a certain genetic background makes individuals more (or less) susceptible to the environment, for better or for worse (Belsky and van IJzendoorn, 2017). Research on differential susceptibility is also integrated in the human cohorts. So far, this field has predominantly focused on serotonergic and dopaminergic polymorphisms and factors involved in amine neurotransmission (Assary et al., 2018; Bakermans-Kranenburg and van IJzendoorn, 2015). The second set of experiments (described in Section 2.2) was carried out in a complex environment to which rats were exposed from early adolescence onwards, with continuous presence of many peers and challenges in the home cage. In Section 3, we highlight the behavioral tests that are currently available to investigate social behavior and behavioral control in rodents. Some new tests were developed within the framework of CID, to improve alignment with the experimental endpoints in humans. Fig. 1 shows an overview of the designs of the rat and mouse studies we have performed and Section 3.2 describes the results we have obtained so far. Finally, in Section 4 we summarize the main observations obtained in the CID rodent cohort to date and discuss their added value to the investigations in human cohorts of the consortium.

## 2 Rearing conditions used in the CID animal cohort

Even more so than in humans, the mother embodies and regulates a large part of the environment of a rodent pup in its first period of development. Not only does she provide milk, but she regulates the temperature in the nest, defends the nest against intruders and provides thorough licking and grooming of the pups needed to defecate and urinate, and to stimulate growth hormones (Cummings et al., 2010). Interfering with this maternal behavior by taking the mother away from the nest, or making her life more difficult or easy, will influence the early life environment of the pups and hence their development (Bonapersona et al., 2019; Chen and Baram, 2016; Curley and Champagne, 2016; Krugers and Joëls, 2014; Peña et al., 2019). We can easily see the parallel with the human situation, in which a difficult and demanding environment for caretakers can impact on child development and wellbeing (Belsky and De Haan, 2011; Cabeza de Baca and Ellis, 2017; Glynn and Baram, 2019; Heim and Binder, 2012; Herzberg and Gunnar, 2019).

### 2.1 Models aimed at intervening with mother-pup interactions

In the CID rodent cohort, we have extensively studied effects of early postnatal interventions. Although many models are available, we restricted ourselves to a limited number of well-defined variants, which are described in more detail below.

We used both rats and mice, we carefully selected the species that was most optimal for each of the test we performed. For the behavioral endpoints in the CID consortium, rats were the more obvious animal of choice, since they show a more extensive social behavior repertoire compared to mice and display robust sociability toward members of their own group (Kondrakiewicz et al., 2019). We have chosen to work with the Wistar rat, a commonly used laboratory rat. Conversely, to exert control over genetic background and study gene-environment interactions, we had more possibilities and experience with mice and we used two heterozygous knock-out models: the mineralocorticoid receptor (MR) knock-out (Berger et al., 2006) for which breeding was already established in our lab; and a newly purchased dopamine receptor-4 (DRD4) knock-out mouse line (Rubinstein et al., 1997). Both lines were back-crossed on a C57Bl/6 inbred mouse line. The mineralocorticoid receptor has a high affinity for cortisol (corticosterone in rodents) and is thought to be implicated in the effect of sensitive parenting on attachment security in humans (Luijk et al., 2011). Moreover, polymorphisms in the MR gene have been found to enhance the negative effects of childhood maltreatment (Vinkers et al., 2015) or neglect (Bogdan et al., 2012). Next to this, a variant in the dopamine D4 receptor (DRD4−7R, exon III 7-repeat), which is associated with reduced efficacy, is a candidate gene in human studies on differential susceptibility. Individuals carrying the 7-repeat version of the gene, are at increased risk to develop externalizing problems after experiencing parental insensitivity (Bakermans-Kranenburg and van IJzendoorn, 2006; Windhorst et al., 2015). Conversely, an intervention aimed at promoting positive parenting and sensitive discipline was found to be more effective in children carrying this DRD4−7R variant of the gene (Bakermans-Kranenburg et al., 2008).

#### 2.1.1 Rat early life model: Maternal deprivation for 24 h at postnatal day 3

Deprivation of maternal care for 24 h, or repeatedly (models range from 3 to 12 h) in the first two weeks of a pup’s life leads to a surge in corticosterone and HPA-axis activity in a period where circulating hormone levels are normally low (Sapolsky and Meaney, 1986). This corticosterone overexposure is known to alter HPA-axis functionality and stress reactivity later in life (Van Bodegom et al., 2017) and has been shown to cause deficits in cognitive and emotional behavior (Krugers and Joëls, 2014; Lupien et al., 2009; Marco et al., 2015). Rodents have an early timing of birth (a more altricial species) compared to humans, which should be taken into account when comparing pre and postnatal brain development. Although the first 10 days of rodent postnatal life are thought to coincide with human brain development in the last trimester based on gross brain development (Semple et al., 2013; Workman et al., 2013), a direct comparison of the perinatal development between species is more complicated, since the maturing brain is more profoundly affected by activity and environmental influences after birth compared to prenatally (Clancy et al., 2001; Semple et al., 2013). The model of 24 h deprivation on postnatal day 3, that aims to model neglect or trauma in young children, was used in several earlier studies in our lab, with thoroughly examined HPA-axis changes (Enthoven et al., 2010; Schmidt et al., 2004; Workel et al., 2001) and long lasting structural (Loi et al., 2014; Oomen et al., 2009; Sarabdjitsingh et al., 2017) and functional changes in the brain (Derks et al., 2016; Loi et al., 2017; Oomen et al., 2011, 2010). *Procedure:* We used this model in Wistar rats, mothers in the deprived group were transferred to a clean cage on postnatal day 3 and kept in this cage for 24 h. The litter was weighed, culled to a minimum of 6 and a maximum of 10 pups and when necessary litters were sex balanced before returning to the home cage (each litter contained a minimum of n = 3 of each sex per nest and a balance of 5/5 or 4/6 for nests containing 10 pups). Pups in the deprived group were transferred to an adjacent room where the cage was placed on a heating pad to prevent hypothermia. In the control group, the dam and litter were returned to the home cage within 2 min. Pups in the deprived group were thus deprived from milk, maternal contact and maternal licking and grooming. After 24 h, the litter was taken back to the original room and the mother was reunited with her litter and left undisturbed -apart from partially refreshing the sawdust once a week- until weaning. This model should not be confounded with social isolation, where individual pups are kept apart, and are therefore also deprived of contact with littermates.

#### 2.1.2 Mouse early life model: Limited nesting/bedding material and communal nesting of 2 dams

Aiming to provide a harsh versus an enriched early life environment, we exposed mice (dams with their litter) to either a cage with limited bedding on a grid floor and minimal nesting material (Rice et al., 2008), or a communal nesting setting with two dams together in a cage (Branchi et al., 2006). The limited nesting and bedding model induces fragmented care and mimics chronic early life stress, affecting HPA-axis (Rice et al., 2008) and leading to long-term effects on cognition, metabolism and emotional behavior (Davis et al., 2017; Molet et al., 2016; C.-D. Walker et al., 2017a, 2017b). This unpredictable pattern of maternal behavior has also been postulated as a risk factor for child and adolescent psychopathology (Glynn and Baram, 2019). Communal nesting is an early social enrichment and has been shown to stimulate a more elaborate social and emotional behavior in adulthood (Branchi et al., 2010, 2006; Branchi and Alleva, 2006). *Procedure:* The experimental conditions (limited nesting or communal nesting) started at postnatal day 2, when all litters were weighed and culled to 6–7 pups, with each litter containing a minimum of 2 pups of each sex. Both conditions were compared to the “standard nesting” condition, i.e. a single breeding dam in a cage with a standard amount of bedding and nesting material. The litter of the dam that was coupled with an experimental litter to form a communal nest, was marked with a non-toxic surgical marker on postnatal days 2 and 6 to ensure correct allocation of the pups to either mother at the end of the communal nesting condition. The communal nesting or limited bedding and nesting condition lasted for 7 days in the MR^+/−^ study and 12 days in the DRD4^+/−^ study. At postnatal day 9 or 14 respectively, all pups returned to standard nesting conditions until weaning at postnatal day 21.

### 2.2 A model intervening with rearing environment from early adolescence onwards

Housing in cages with obstacles, shelters, running wheels and several cage mates has been generally considered to be an enriched environment (Würbel, 2001) and has been shown to increase brain plasticity (Gelfo, 2019; Simpson and Kelly, 2011; van Praag et al., 2000v). This is of course contrasted against the standard laboratory setting, which is a low-stimulating environment with only 2–4 animals in a small cage, food ad libitum and a wooden block to chew on. We started off with the idea that exposure to a more naturalistic “enriched” environment during adolescence, a period in which brain circuitry implied in social behavior is still in development (Casey et al., 2019; Fuhrmann et al., 2015; Vijayakumar et al., 2018), might be able to counteract the negative effects of early postnatal stress on various aspects of social behavior (Cui et al., 2006; Francis et al., 2002; Kentner et al., 2019; Morley-Fletcher et al., 2003; Vivinetto et al., 2013). In rats, the period directly after weaning (postnatal day 21 to around postnatal day 30) is considered to reflect the prepubertal/early adolescent period, which is followed by a period (day 30–60) in which rats undergo the behavioral and neurobiological transformations associated with adolescence (Eiland and Romeo, 2013; McCormick and Mathews, 2010; Spear, 2000), reaching (young) adulthood at postnatal day 60.

**Fig. 2:**
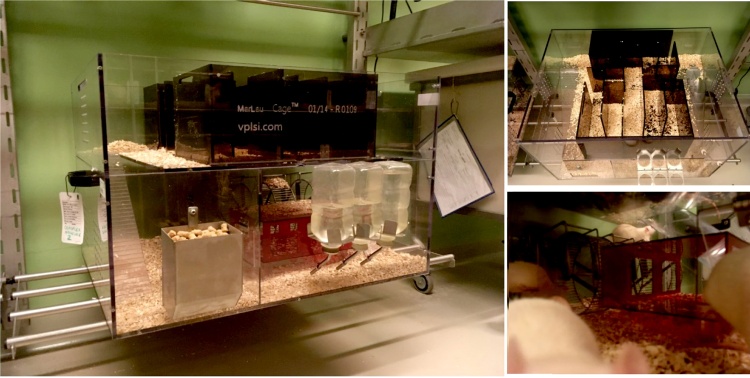
Complex housing in Marlau™ cages (Viewpoint, Lyon, France), housing 10-12 same-sex rats per cage. Marlau cages (60 × 80 × 51 cm) have 2 levels: the first level contains a big compartment with three running wheels, a shelter, ad libitum access to water, 2 woodblocks, and a climbing ladder to the second lever, where a maze has to be passed to gain access to a tube leading to the food compartment on the first level.

An “enriched” setting consists of many different aspects, like exercise, social contact and challenges. Our goal was not to distinguish these different aspects, but rather to consider the setting as a relatively naturalistic, complex and challenging environment that can positively influence the development of social skills (Gubert and Hannan, 2019; Würbel, 2001). Considering the existing studies at the time that CID was started, a certain lack of standardization in “enriched” settings was apparent, each lab having constructed its own setting which makes comparison of studies across labs difficult (Simpson and Kelly, 2011). This is why we chose to work with the Marlau cages (Fig. 2), in which rats can express much of their naturalistic behavior and which can be purchased by every lab (Fares et al., 2013). *Procedure:* In our design, animals were housed in these cages with 10 same-sex playmates from postnatal day 26 onwards (early adolescence) until (and during) behavioral testing at 3–4 months of age. To provide cognitive stimulation, the maze in the upper compartment was changed twice a week in its configuration. Rats could freely move in both the upper and lower compartment tumbling down or by using stairs and tunnels, and had access to running wheels, a shelter place and a food and water compartment.

Observing the behavior of rats that grew up in these cages, we have gradually rephrased the term “enriched” into “complex” environment. The reason is that, next to being able to express a more complete behavioral repertoire in these cages, this environment is also more demanding in terms of adaptation to a changing setting and living in a colony where there is limited space to retract. As such it could model our rapidly changing and crowded (human) society, in which social skills are paramount to thrive (D. M. Walker et al., 2017a, 2017b). In the more recent series of experiments, we used this setting as an early life condition by itself, rather than an intervention to (potentially) normalize the effects of early life adversity.

## 3 Rodent behavioral tests in CID: measuring social competence in rodents

Social behavior refers to a realm of behaviors all oriented towards interaction with conspecifics. Being socially competent implies behaving in socially accepted ways, showing behavior that is understood by others and showing a correct amount of behaviors like play, curiosity, aggression, caring for others or acting together, to be able to cope with different situations, defend one’s own integrity or offspring’s survival and thrive in society (Caldwell and Elliott Albers, 2016; Hofmann et al., 2014; Seebacher and Krause, 2019; Taborsky and Oliveira, 2012). This is true for both humans and rodents and underlying neural mechanisms appear to be conserved (Burkett et al., 2016; Donaldson and Young, 2008). Although there is an ongoing debate whether or not empathy is a typically human behavior and whether it is really selfless, there are strong theories that there are many (pro)social behaviors that compose the building blocks on which empathy was able to evolve (Chen, 2018; De Waal and Preston, 2017).

### 3.1 Systematic review of literature on early life models and adult social behavior

We first considered the variety of tests available in the social domain. Most studies on the effects of early life adversity in fact focus on later life learning, memory, anxiety and stress reactivity. When we started out in 2013, not many studies had been published on the effect on social behavior. This was not very surprising since social competence is largely understudied in rodents, hence the need for more tests on (pro)- social behavior. For the purpose of this review, we have updated the search on early life adversity and social behavior, using a systematic review approach (see Table 1). The details of the method were recently described by Bonapersona et al. (2019), and have now been extended with a specific focus on social behavior.

**Table 1:** Results of a systematic literature search conducted on the effects of early life adversity (ELA) on adult social behavior, arranged according to the experimental endpoint. *If “no”, the mothers were purchased pregnant, which represents an additional early life event. ** Maternal separation (MS) includes separation from the mother between 3 and 6 h daily, maternal deprivation (MD) is a separation from the mother of either one time 24 h or 12 h for two consecutive days, “isolation” means separation from both mother and siblings, between 3 and 6 h daily. P = postnatal day, / (slash) separates different independent experiments with comparable procedures, NS = not specified. *** Refers to phase of circadian cycle at the time of testing. ‘↓’ decreased, ‘↑’ increased or ‘=’ no change.

	**Species, Strain**	Own breeding?*	ELA model**	**Sex**	**Total sample size** **(ELA + control)**	Test Phase***	**Result**
**Social interaction**							
Bahi (2016)	Rats, Wistar	yes	MS, P1-P11	Males	20	Light	**↓**
Lukas et al. (2011)	Rats, Wistar	yes	MS, P1-P14	Males	20	Light	**=**
Rincel et al. (2016)	Rats, Wistar	yes	MS, P2-P14	Males	14	Light	**↓**
Rincel et al. (2019)	Rats, Wistar	yes	MS, P2-P14	Males	21/21	Light	**=** / **=**
Zugno et al. (2013)	Rats, Wistar	no	MS, P1-P10	Males	12	NS	**=**
Mehta and Schmauss (2011)	Mice, Balb C	yes	MS, P2-P15	Males	16	Light	**=**
Mehta and Schmauss (2011)	Mice, Balb C	yes	MS, P2-P15	Females	16	Light	**=**
Mehta and Schmauss (2011)	Mice, C57Bl/6	yes	MS, P2-P15	Males	16	Light	**=**
Mehta and Schmauss (2011)	Mice, C57Bl/6	yes	MS, P2-P15	Females	16	Light	**=**
Takase et al. (2012)	Rats, Wistar	yes	MD, P9-P11	Males	24	Light	**↓**
Zamberletti et al. (2012)	Rats, Sprague Dawley	no	MD, P9-P10	Males	19	Light	**=**
Zamberletti et al. (2012)	Rats, Sprague Dawley	no	MD, P9-P10	Females	19	Light	**=**
Bouet et al. (2011)	Mice, NMRI	yes	MD, P9	Males	29	NS	**=**
Bolton et al. (2018)	Rats, Sprague Dawley	no	Limited bedding, P2-P9	Males	11	Light	**=**
Yan et al. (2017)	Rats, Long Evans	yes	Limited bedding, P8-P12	Males	14	Light	**↓**
Heun-Johnson and Levitt (2018)	Mice, C57Bl/6	yes	Limited bedding, P2-P9	Males	32	Light	**↓**
van der Kooij et al. (2015)	Mice, C57Bl/6	yes	Limited bedding, P2-P9	Males	28	Light	**↓**
van der Kooij et al. (2015)	Mice, C57Bl/6	yes	Limited bedding, P10-P17	Males	28	Light	**↓**
Bahi (2017)	Rats, Wistar	yes	Isolation, P1-P11	Males	20/20	Light	**↓** / **↓**
Kambali et al. (2019)	Rats, Wistar	no	Isolation, P4-P14	Males	26/26	Light	**=** / **↓**
Mintz et al. (2005)	Rats, Wistar	yes	Isolation, P1-P14	Males	8/8	Dark	**=** / **=**
Ohta et al. (2019)	Rats, Sprague Dawley	no	Isolation, P2-P20	Males	30	Light	**=**
Toth et al. (2008)	Rats, Sprague Dawley	yes	Isolation, P5-P7	Males	20/20	Light	**↓**/ **=**
Uribe et al. (2016)	Rats, Sprague Dawley	yes	Isolation, P2-P14	Males	10	Light	**↓**
Arnett et al. (2015)	Mice, C57Bl/6	no	Isolation, P1-P14	Males	26	Light	**↑**
Sachs et al. (2015)	Mice, C57Bl/6	yes	Isolation, P2-P12	Males	44	NS	**=**
Sachs et al. (2015)	Mice, C57Bl/6	yes	Isolation, P2-P12	Females	44	NS	**=**
Shin et al. (2018)	Mice, C57Bl/6	unclear	Isolation, P1-P14	Males	12	Light	**↓**
Starr-Phillips and Beery (2014)	Rats, Long Evans	yes	Licking/Grooming, P1-P6	Males	30	Light	**↓**
Starr-Phillips and Beery (2014)	Rats, Long Evans	yes	Licking/Grooming, P1-P6	Females	30	Light	**↓**
Starr-Phillips and Beery (2014)	Rats, Long Evans	yes	Licking/Grooming, P1-P6	Females	29	Light	**=**

**Three chambers test: social interest**							

Benetti et al. (2009)	Rats, Wistar	yes	MS, P1-P10	Males	20	Light	**↓**
Hulshof et al. (2011)	Rats, Wistar	yes	MS, P2-P15	Males	16/16	Light	**=** / **=**
Karen and Rajan (2019)	Rats, Wistar	unclear	MS, P5-P10	Males	22	Light	**=**
Bondar et al. (2018)	Mice, C57Bl/6	yes	MS, P2-P14	Males	17	Light	**=**
Bondar et al. (2018)	Mice, C57Bl/6	yes	MS, P2-P14	Females	19	Light	**=**
Zoicas and Neumann (2016)	Mice, CD1	yes	MS, P1-P14	Males	24	Light	**=**
Cabbia et al. (2018)	Rats, Wistar	yes	MD, P11-P12	Males	29/29	Light	**=** / **=**
Cabbia et al. (2018)	Rats, Wistar	yes	MD, P11-P12	Females	29/29	Light	**=** /**↓**
Kentrop et al. (2018)	Rats, Wistar	yes	MD, P3-P4	Males	32	Light	**=**
Kentrop et al. (2018)	Rats, Wistar	yes	MD, P3-P4	Females	32	Light	**=**
Kohl et al. (2015)	Mice, C57Bl/6	yes	Limited bedding, P2-P9	Males	20/30	Light	**=** / **=**
Ohta et al. (2019)	Rats, Sprague Dawley	no	Isolation, P2-P20	Males	30	Light	**=**
Ohta et al. (2019)	Rats, Sprague Dawley	no	Isolation, P2-P20	Males	48	Light	**=**
Harrison et al. (2014)	Mice, C57Bl/6	yes	Isolation, P1-P14	Males	29	Light	**=**
Shin et al. (2018)	Mice, C57Bl/6	unclear	Isolation, P1-P14	Males	18	Light	**↓**
Wang et al. (2017)	Mice, C57Bl/6	yes	Isolation, P2-P20	Males	14	Light	**↓**

**Three chambers test: social discrimination**							

Benetti et al. (2009)	Rats, Wistar	yes	MS, P1-P10	Males	20	Light	**↓**
Hulshof et al. (2011)	Rats, Wistar	yes	MS, P2-P15	Males	16/16	Light	**=** / **=**
Karen and Rajan (2019)	Rats, Wistar	unclear	MS, P5-P10	Males	22	Light	**=**
Lukas et al. (2011)	Rats, Wistar	yes	MS, P1-P14	Males	20/20/17	Light	**=** / **↓** / **=**
Zoicas and Neumann (2016)	Mice, CD1	yes	MS, P1-P14	Males	24	Light	**=**
Kentrop et al. (2018)	Rats, Wistar	yes	MD, P3-P4	Males	32	Light	**↓**
Kentrop et al. (2018)	Rats, Wistar	yes	MD, P3-P4	Females	32	Light	**↓**
Alteba et al. (2016)	Rats, strain NS	no	Limited bedding, P9-P14	Males	16	Light	**↓**
Alteba et al. (2016)	Rats, strain NS	no	Limited bedding, P9-P14	Females	16	Light	**↓**
Kohl et al. (2015)	Mice, C57Bl/6	yes	Limited bedding, P2-P9	Males	20/30	Light	**↓** / **=**
Ohta et al. (2019)	Rats, Sprague Dawley	no	Isolation, P2-P20	Males	48	Light	**↓**
Zhang et al. (2014)	Rats, Sprague Dawley	yes	Isolation, P1-P21	Males	15/24	Light	**=** / **↑**
Harrison et al. (2014)	Mice, C57Bl/6	yes	Isolation, P1-P14	Males	29	Light	**↓**

**Aggressive behavior**							

Frank et al. (2019)	Rats, Sprague Dawley	yes	MS, P2-P20	Males	58	Dark	**↑**
Hidaka et al. (2018)	Mice, C57Bl/6	yes	MS, P2-P14	Males	18	Dark	**=**
Weidner et al. (2019)	Rats, Wistar	yes	MS, P2-P15	Males	18	Light	**=**
Zamberletti et al. (2012)	Rats, Sprague Dawley	no	MD, P9-P10	Males	19	Light	**=**
Zamberletti et al. (2012)	Rats, Sprague Dawley	no	MD, P9-P10	Females	19	Light	**↑**

**Social fear conditioning**							

Zoicas and Neumann (2016)	Mice, CD1	yes	MS, P1-P14	Males	17	Light	**=**

An updated search (compared to Bonapersona et al., 2019) was conducted on Nov. 20th 2019 on the electronic databases PubMed and Web of Science, leading to 319 new unique publications; based on the title and abstract, articles were excluded only if the experiments were not conducted in rodents (mice and rats) and/or did not use an early life adversity model. The search string can be found in the Supplementary Methods. Early life adversity was defined as postnatal alteration of maternal care, and could be described by the following models: maternal separation/deprivation, isolation, limited nesting and bedding, and natural licking and grooming. Next, full text was examined and experiments were included if they met the predefined inclusion criteria; specifically, social behavior should have been investigated in adulthood (>8 weeks of age, younger than 1 year). Ultimately, 12 new publications were included from the updated search, thereby reaching a total of 38 articles that investigated the effects of early life adversity on social behavior, leading to the 67 comparisons in Table 1. To limit subjectivity in the data gathering process, records were catalogued in a standardized database. Summary statistics information (sample size, mean, standard deviation or standard error of the mean) were extracted from text (preferred if applicable) or figures (Web Plot Digitalizer). If it was not specified whether figures reported standard deviations or standard error of the means, we conservatively assumed that they were standard errors. A Welch *t*-test (two-sided, α = 0.05) was performed on summary statistics to evaluate whether each comparison between a control and an experimental group was significantly different. The conclusions might therefore slightly differ from the original publications, where groups might have belonged to more complex experimental designs.

As expected, the majority of papers (69 %, taking both rat and mouse studies into account) were published after the start of CID in 2013. Most experiments were performed on males (84 %), however, no obvious sex differences seem to appear in the ELA effect on the measured social behavior. The ELA conditions that are represented most in this overview are the models of Maternal Separation (39 % of papers), where the mother is taken away from the pups on a daily basis for 3−6 hrs and Isolation (31 % of papers), where pups are separated from both mother and siblings, also between 3 and 6 h daily. These are followed by the limited bedding and nesting model (15 % of papers), a more recently developed model where mothers and pups are put into austere housing conditions and Maternal Deprivation (13 % of papers), where the mother does not have access to the pups for either a continuous period of 24 h or for 12 h on two consecutive day. Only 1 paper reports on the effects of natural variation in licking and grooming on social behavior. Overall, 62 % of comparisons between ELA and control animals on social behavior was found the be non-significant, with slight differences between tasks and ELA models.

Regarding the social tasks performed in adulthood, free social interaction was analysed most often; here, a test animal can interact with a non-familiar conspecific control animal in a neutral setting. This control animal is generally younger, to avoid aggressive encounters. Approximately half of the studies found a decrease in social interaction in this task after ELA, the other half did not find effects. Social interest and social discrimination in the three chamber task is also regularly selected as a behavioral outcome. The advantage of this task is that the test animal will initiate social contact, by choosing to enter the chamber in which a non- familiar rat is present in a confined space. A decrease in social interest (i.e. approaching a confined rat) was only observed in 17 % of comparisons, while half of the studies found a decrease in social discrimination (making a difference between a new and a previously encountered animal) in ELA animals. Interestingly, social discrimination does not only reflect social abilities but also the mnemonic ability of remembering the previously encountered conspecific. Only 4 studies measured aggressive behavior, of which 3 used the resident-intruder task, where a non-familiar rat is introduced in the home-cage. Also for this task results were mixed (non-significant and increases in aggression in the ELA animals). Overall, ELA does not seem to have a substantial effect on adult social interest; but it does affect social interaction measures and social discrimination in approximately half of the studies. The differences in outcome between studies might be the result of differences in conditions across labs (Voelkl et al., 2020). Although similar protocols are used, differences in husbandry and other experimental conditions in the stables might affect the impact of these early life models. One aspect that we have taken into account is whether the breeding was done in the own facility, or that pregnant females were purchased form a provider (see column “Own breeding”). This latter situation represents an extra stressor for the pregnant females, that might represent an extra early life adversity adding to a vulnerable phenotype (Bonapersona et al., 2019; Daskalakis et al., 2013; McEwen, 1998). Regarding social behavior, we did not observe more clear-cut results of ELA in studies using dams from a provider versus in-house breeding, but control litters were of course also exposed to this prenatally induced stressor. We are of the opinion that these types of conditions are important to take into account, and breeding and housing conditions should be reported as extensively as possible to be able to compare studies.

Not surprisingly, most studies were performed in rats (64 % of papers). In both species, the overall pictures emerges that the effect of both maternal separation and maternal deprivation on social behavior is not significant in most cases, although some rat studies report a decrease in social behavior and an increase in aggression. In mice, none of the studies using these models showed significant differences in social behavior. For the isolation model in both rats and mice, around half of the studies showed a decrease in social behavior and half gave non-significant results. The limited bedding and nesting model seems to affect social behavior in both rats and mice most consistently, with six comparisons showing a decrease in social behavior and 3 non-significant studies.

### 3.2 Our early life models and social competence outcome

The maternal deprivation model we used in rats, with 24 h deprivation on postnatal day 3, has not been used in other studies on social behavior. Despite this fact, our results appear to be in line with other deprivation and separation models, that have found limited effects of maternal deprivation on social interest, yet show a decrease in social discrimination (see Table 1). We originally used the complex housing condition as a possible condition that could rescue the early life efffects. Based on the promising effects of the limited bedding and nesting model and the lack of effects in other mice models, we have chosen this model as a negative early life environment in our mouse experiments, next to communal nesting as an enriched early environment. We focused on the effects of these early life conditions on the timing of sexual maturation (puberty onset) and maternal care towards own offspring, which has not been tested before. For the rat studies, we chose certain social tasks from the existing literature that seemed promising and relevant for the social domain. These comprised tasks addressing social play during adolescence (a developmental period not included in Table 1) and social exploration/social memory in adulthood. Since none of the existing tasks captured the element of pro-sociality, we adapted and redesigned two pro-social lever pressing tasks, i.e. a two-choice task for sucrose reward and a motivation task to liberate a trapped cage mate. The tests that we selected for the rodent cohort are described in brief in section 3.2.1 (rat social behavior), 3.2.2 (mice social behavior) and 3.2.3 (rat behavioral control). Full details on each task can be found in the respective papers (Kentrop et al., 2018, [Kentrop et al., 2016] 2016, Kentrop et al. this issue; Knop et al., 2020, [Knop et al., 2019] 2019; van der Veen et al., 2015). The results we have obtained in these tasks are summarized in Section 4 and Table 2, Table 3.

**Table 2:** Summary of our rat studies: Main effects of maternal deprivation and complex housing compared to their respective control conditions. ‘↓’ decreased/impaired, ‘↑’ increased/improved or ‘=’ no change. MD*CH: interaction effect of maternal deprivation (MD) and complex housing (CH) in females (F) and males (M). Because of lengthy behavioral procedures, not all tests could be performed in both males and females (marked with −). For each paper, studies were performed with different batches of animals. Rats were either isolated for 3h or 24h before a play session. The grey numbers in superscript refer to the studies that report the results: ^1^van der Veen et al. 2015, ^2^Kentrop et al. 2016, ^3^Kentrop et al. 2018, ^4^Kentrop et al, this issue.

Experimental readouts		Maternally deprived (MD) versus non-deprived		Complex housing (CH) versus Standard housing		MD*CH
		Females	Males	Females	Males	F/M
Adolescence	Bodyweight	↓^3^	↓^1,2,3^	=^3^	↓^1,3^	no^1,2,3^
	Social play (3 h iso)	=^3^	=^3^	↓^3^	↓^3^	no^3^
	Social play (24 h iso)	=^3^	↓^3^	↑^3^	↑^3^	no^3^

Adulthood	Bodyweight	↓^3^	=^1,3^/↓^2^	↓^3^	=^1,3^	no^1,2,3^
	Behavioral inhibition	–	=^1^/↓^2^	–	↓^1^	no^1,2^
	Attention	–	=^1,2^	–	↑^1^	no^1,2^
	Social interest	=^3^	=^3^	=^3^	↓^3^	no^3^
	Social discrimination	↓^3^	↓^3^	=^3^	=^3^	no^3^
	Pro-social decision making	–	–	–	↓^4^	–
	Motivation for pro-social liberation	–	Work in progress	–	Work in progress	–

**Table 3:** Summary of our mouse studies: Effects of three different early life housing conditions (post-hoc tests performed after main effects of nesting condition) and main effects of genotype (MR^+/−^ and DRD4^+/−^ versus the respective control wildtype mothers). ‘↓’ decreased/delayed, ‘↑’ increased/accelerated or ‘=’ no change. G*E: gene-environment interaction in the effect of limited nesting and communal housing on licking/grooming in DRD4^+/−^mice. The grey numbers in superscript refer to the studies that report the results: ^1^Knop et al. 2019, ^2^Knop et al., 2020.

Experimental readouts		Limited nesting versus Standard nesting		Communal nest versus Standard nesting		Limited nesting versus Communal nest		Geno type *MR^+/−^*	Geno type *DRD4^+/−^*
		Females	Males	Females	Males	Females	Males	F/M	F/M
Adolescence	Bodyweight	=^1^/↓^2^	↓^1,2^	=^1,2^	=^1,2^	↓^1,2^	↓^1,2^	=/=^1^	=/=^2^
	Puberty onset	=^1^/↓^2^	↓^1,2^	=^1,2^	=^1,2^	=^1^/↓^2^	↓^1,2^	=/=^1^	=/=^2^

Adulthood-Maternal care	Arched-back nursing	=^1^/↓^2^	–	=^1,2^	–	=^1^/↓^2^* *p = .057	–	↓^1^	=^2^
	Passive nursing	=^1,2^	–	=^1,2^	–	=^1,2^	–	↑^1^	=^2^
	Total nursing	=^1,2^	–	=^1,2^	–	=^1^/↓^2^	–	=^1^	=^2^
	Licking/grooming	=^1,2^	–	=^1,2^	–	=^1^/↓^2^	–	=^1,2^	G*E
	Fragmentation	=^1,2^	–	=^1,2^	–	=^1,2^	–	↑^1^	=^2^
	Unpredictability (on the nest)	=^1,2^	–	=^1,2^	–	=^1^/↑^2^	–	=^1^	=^2^
	Time on nest	=^1^/↓^2^	–	=^1,2^	–	=^1^/↓^2^	–	=^1^	=^2^
	Pup retrieval	=^1,2^	–	=^1,2^	–	=^1,2^	–	=^1^	=^2^

In some of our experiments, we tested animals during the light phase of the day-night cycle, under dim light conditions (adolescent social play and the 3-chamber task); this agrees with common practice, i.e. only 3 studies reported in Table 1 took place during the dark phase. The pro-social tasks and 5-choice serial reaction time task, however, were performed during the dark phase of the day/night cycle. These tasks require considerable effort of the rats which is more natural to happen during the active (dark) phase of the cycle. Maternal care was observed both during the light and dark phase.

#### 3.2.1 Social competence measured in our rat models

##### 3.2.1.1 Social play in young animals (outcome in young adolescent rats)

Play behavior, also referred to as play fighting, is a characteristic behavior observed in young adolescent animals and one of the first social behaviors that does not include interaction with the mother. It is conceived as a precursor of adult social, aggressive and sexual behavior, where boundaries are explored and social rules are learned (Pellis and Pellis, 2017; Vanderschuren et al., 2016). In children there is an equivalent behavior of pretend play and rough-and-tumble play (Lillard, 2017; Palagi et al., 2016). Social play occurs spontaneously but can be facilitated in a laboratory setting by isolating animals for a short period prior to the actual observation. In rats, the most characteristic play behaviors that are discerned in a play session are pouncing and pinning (Vanderschuren and Trezza, 2014). Pouncing is the initiation of a play bout by approaching and soliciting another animal by touching its neck with the snout; and the subsequent pinning response is discerned when the recipient rolls over to its back and accepts to play (Kentrop et al., 2018; Vanderschuren and Trezza, 2014). *Procedure:* Adolescent testing took place when the animals were between 33 and 42 days of age. Our social play set-up consisted of an arena (40cm × 40cm × 60cm) with 2 cm of wood shavings on the floor. The rats were allowed to habituate to the arena prior to testing. On the day of testing, animals were isolated for either 3 h or 24 h preceding the social encounter in which subsequently play behavior was observed for 15 min. Testing took place during the light phase, under dim light conditions.

##### 3.2.1.2 The three chamber social approach task (outcome in adult rats)

To test social interest in an unfamiliar rat and subsequent discrimination between a recently encountered rat and another unfamiliar rat, we used the three chamber social approach task (Toth and Neumann, 2013). The initiative for social contact has to come from the test rat, since the unfamiliar rats are placed in cylinders within two separate compartments. The cylinders allow for social contact, while preventing aggressive assaults (for more experimental detail see Kentrop et al., 2018). *Procedure:* Animals were habituated to the task in the week before testing. In the social interest phase of the task, an unfamiliar stimulus rat was placed in one of the cylinders, while the other cylinder remained empty. The test rat was allowed to explore the different chambers for 10 min. This was followed by the social discrimination phase, where a new, unfamiliar rat was introduced in the other cylinder and the test rat was allowed to explore the task for another 5 min. In the analysis of this task, the location of the test rat and the time a rat spends around each cylinder can be tracked automatically. This test reveals both interest in social contact and social memory, i.e. the extent to which other rats are remembered. The three chamber task was performed during the light phase under dim light conditions.

##### 3.2.1.3 (Pro)-social decision making: Two-choice task pressing for sucrose reward

With this task we intended to assess whether an action to obtain something valuable can be guided by the consequences it has for another individual. We developed a task based on existing pro-social (two)-choice tasks (Marshall-Pescini et al., 2016). In our version, a test subject has to perform an act to earn a sucrose reward and it has two options to do so: One option leads to a reward for only the test rat, while the other option leads to a reward for both the test rat and the partner rat. This partner rat is located in an opposite compartment separated from the test rat by a transparent wall with small holes that allow smelling the other rat but no physical contact. The test rat has to press one of two levers several times to obtain a sucrose reward. One lever dispenses a sucrose pellet only to the test rat; the other lever dispenses pellets to both the test and the partner rat. Through the transparent wall, the test rat can observe which lever is coupled to a reward for both and for which lever it will be the only one receiving a reward (Kentrop et al., 2020). *Procedure:* In this task, animals are slightly food deprived (90–95 % of free fed bodyweight) to increase their motivation to obtain the sucrose pellets. Animals were habituated to the task and trained to perform first on a fixed ratio 1 (FR1) and then on an FR3 schedule of reinforcement (i.e., in which 3 lever presses are needed to obtain a sucrose reward). Training required approximately a week and subsequent testing another month. Testing took place during the dark phase of the light cycle.

##### 3.2.1.4 Acting (pro)-socially: Motivation to liberate a trapped cage-mate

**Fig. 3:**
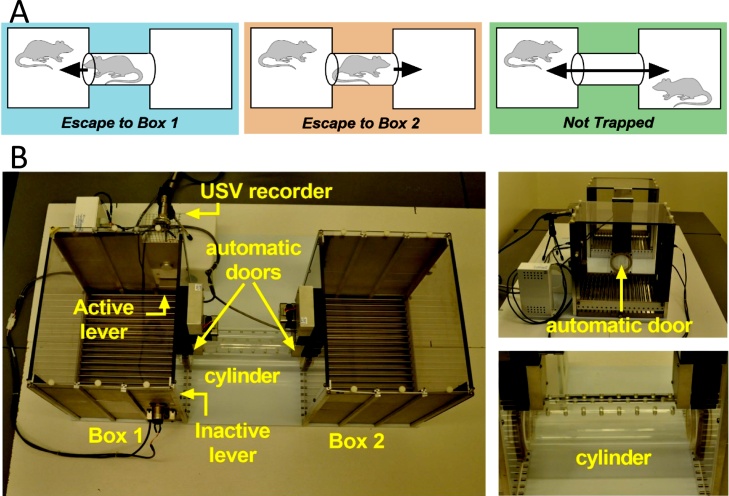
The liberation task set-up. (A) Three different configurations can be tested: 1) *Escape To Box 1* in which the partner is trapped in the cylinder and can be liberated from the cylinder into box 1, which is the test rat compartment, 2) *Escape To Box 2* in which the partner is trapped in the cylinder and can be liberated into box 2, and 3) *Not Trapped configuration* in which the partner is situated in box 2 and lever presses of the test rat opened both doors. (B) Liberation task set-up: 2 operant chambers are connected through a removable cylinder (chambers 29.5 × 23.5 × 27.3 cm, cylinder 25 cm in length and 7.5 cm in diameter, Med Associates, St. Albans, VT, USA). The cylinder can be closed on both sides by automated mechanical doors made of transparent Plexiglas with holes (6 mm in diameter) that allowed rats to see, smell, hear but not touch each other.

In this final task we assessed the motivation of a rat to perform a pro-social action without directly gaining something in return. As the basis for our task we used the set-up from Bartal et al. (Bartal et al., 2011), where a partner rat that is trapped in a cylinder can be liberated by the test rat. In our setting, instead of liberating the partner by tilting over the cylinder door, the test rat had to press a lever which automatically opened the door and liberated the trapped partner rat. We designed a protocol where progressively more effort (and thus more motivation) is needed to open the door and liberate the trapped rat. Next to the pro-social act of freeing a trapped partner, social contact can be an important driver to liberate a partner rat. To examine this aspect, we also allowed rats to open the door towards a separate compartment, away from the test rat (see Fig. 3). *Procedure:* In order to acquire lever pressing before the start of the liberation task, rats were trained for a week with sucrose rewards in separate operant boxes. Animals were habituated to the liberation task and trained to perform first on an FR1 and then on an FR3 schedule of reinforcement (i.e., in which 3 lever presses are needed to open the cylinder door). Training took around a week and subsequent testing another month. Testing took place during the dark phase of the light cycle. Analyses of these experiments are still ongoing.

#### 3.2.2 Social competence measured in our mouse models

We have extensively looked at the maternal (social) behavior of mouse dams that were exposed to different early life environments as described in section 2. Maternal care was observed in the home cage during the early postnatal period. Moreover, to probe maternal responsiveness, a pup retrieval test was performed (Rosenblatt, 1967). In this test, pups are scattered in the home cage by the experimenter, and the latency to retrieve all pups by the dam is measured. *Procedure:* Dams were observed three times per day during the early postnatal period from postnatal day 2–9. Two observation periods were done in the light phase and one in the dark phase, under red light conditions. Each observation period lasted 75 min. We used the so-called intermittent schedule of observation (Liu et al., 1997), i.e. the behavior of the rats was observed every 3rd minute, resulting in 25 observations per observation period and 75 observations per day (see (Knop et al., 2019) for more experimental detail). A pup retrieval test was realized on postnatal day 7.

We were not only interested in the main maternal behaviors of licking and grooming the pups (LG) and nursing, distinguishing between arched‐back nursing (ABN) and passive nursing (Champagne and Meaney, 2001; Olazábal et al., 2013), but also in the fragmentation (Rice et al., 2008) and predictability of behavior (Molet et al., 2016). These indices reflect respectively the fragmentation in the dam being available for the pups (mother hopping on and off the nest) and the structuring of behavior being predictable (predictability or so-called inverse entropy of behavior). These behaviors can be more directly linked to meta- behaviors in human research, where predictability and structure of parental care are thought to be important for children, and unpredictable behavior favors a negative outcome in terms of neurodevelopment (Glynn and Baram, 2019). Indeed, unpredictable maternal mood and behavior is associated with impaired cognitive and emotional maturation including in the realms of memory and self-control, and with risk for internalizing disorders in children (Davis et al., 2017; Glynn et al., 2018).

#### 3.2.3 Behavioral control measured in our rat models

As a measure of behavioral control, we have studied impulsive action and inhibition of behavior in the 5-choice serial reaction time task (5-CSRTT) (Robbins, 2002). This task is based on the continuous performance task in humans (Cope et al., 2016) and was developed to measure sustained as well as divided attention, both needed to continuously scan 5 holes for the apparition of a stimulus light. *Procedure:* Our rats conducted this task in operant conditioning chambers (MedAssociates, St.Albans, VT, USA) in which experimental contingencies can be controlled and data collected. At first, animals are trained to respond to this stimulus light to obtain a reward (sucrose pellet). In each session, there are 100 trials in a row, where the stimulus light is randomly presented in one of the 5 holes. When the rat pokes its nose into an illuminated hole, a sucrose reward is released into a receptacle facing the 5-choice wall. To collect a reward, the rat thus turns its back to the 5-choice wall. It has to quickly recover the sucrose pellet and turn its attention again to the 5 holes to respond to the next stimulus light. A challenge here is not to miss a stimulus (sustain attention) and to refrain from responding until a next visual stimulus is presented. Anticipatory responses prior to the presentation of the stimulus light are regarded as a measure of impulsivity (Bari et al., 2008). An omission, premature response or incorrect response is punished with a time-out period (reward delay). As reported (Kentrop et al., 2016; van der Veen et al., 2015), all rats performed the task at a high level after intensive training. This is defined as accomplishing 100 trials within 30 min with a high performance accuracy (>80 % correct choice; a mean of 94 % in our studies), low errors of omission (<20; a mean of 9 errors in our studies) and a low number of premature responses (<6 in our studies) (Kentrop et al., 2016; van der Veen et al., 2015). Following the training period, either attentional load can be increased by shortening the stimulus time or introducing a novel object in the cage, or behavioral inhibition is challenged by extending the inter-trial interval or making the interval unpredictable.

## 4 Results with the CID rodent models so far

### 4.1 Maternal deprivation and complex housing models in rats

An overview of all results obtained in our various rat studies is given in Table 2. Overall, our rat studies on maternal deprivation (MD) showed clear effects on bodyweight, i.e. lower weight in adolescence for both deprived males and females (Kentrop et al., 2018, [Kentrop et al., 2016] 2016; van der Veen et al., 2015). During adolescence, social play in the often-used version of depriving the animals of social contact for 3 h prior to the play session (Vanderschuren et al., 2016), was not influenced by MD (Kentrop et al., 2018). However, when animals were deprived of social contact for 24 h before the play session, male rats with a history of MD showed a delayed initiation to play, which was not observed in females (Kentrop et al., 2018). This paradigm of 24 h social isolation might be conceived as more stressful in the deprived animals, since play is initiated in the absence of stress, when the animal is safe, fed and healthy (Vanderschuren and Trezza, 2014). Effects of MD on adult social behavior were evident with regard to social discrimination -which reflects a non-stressful type of learning- but not for social interest, both in males and females (Kentrop et al., 2018). This is in line with our recent meta-analysis (Bonapersona et al., 2019), which showed that adverse conditions experienced in the first 1–3 postnatal weeks are associated with a decrease in non- stressful learning later in life.

[Table 2 here]

We also tested the effects of MD on performance of males in the 5-CSRTT task, in two different studies (Kentrop et al., 2016; van der Veen et al., 2015). Impaired behavioral inhibition was observed only in the study where maternal deprivation also had an effect on adult male bodyweight (Kentrop et al., 2016). Although bodyweight was not related to performance in the task, the early life condition might have had more impact in this first batch of animals. In our meta-analysis (Bonapersona et al., 2019) we found that early life stress combined with other negative experiences (“hits”) is associated with a stronger behavioral phenotype in adulthood than early adversity by itself (single hit). Such multiple hits may e.g. be related to housing conditions that are not controlled for. For instance, in our animal facilities we have a common breeding room where conditions fluctuate, in terms of room usage and experiments running in parallel.

Thus, contrary to our expectations, the MD model we applied in CID did not have a major impact on social behavior and behavioral inhibition. Future studies should perhaps consider a later time point of deprivation which is possibly more critical for the development of brain circuitry involved in social behavior (Casey et al., 2019). Indeed, early life studies including the second postnatal week seem to have more impact on social behavior (Kambali et al., 2019; Peña et al., 2019).

In contrast to the rather subtle effects of MD, the effects of housing in the complex Marlau cages had a major impact on social behavior. What started out as a possible intervention to counteract the effects of MD, appeared to result in a strong phenotype by itself. Complex housing (CH) even had some effects in common with MD, but mostly showed changes in tasks where MD did not. The two interventions never interacted (Table 2). In terms of bodyweight changes, in complex housing (compared to standard housing) males’ bodyweight was reduced in adolescence and females’ bodyweight was decreased in adulthood (Kentrop et al., 2018; van der Veen et al., 2015v). This reduction in bodyweight might reflect the more active life in these cages, with animals being able to move more freely, use the running wheels and have more climbing possibilities. Contrary to the expectation, social play behavior during adolescence was almost non-existing in the standard set-up with 3 h of social deprivation prior to the task. The rats even fell asleep side-by-side during the supposed play session. However, after a more challenging 24 h of social deprivation, both male and female CH animals had a very short latency to start playing and CH males showed more play compared to standard housed animals (Kentrop et al., 2018), which is also observed by others (Morley-Fletcher et al., 2003). Our results may be partly explained by the standard setting in which play behavior is observed, which might be less interesting and play- provoking than the complex home cage. This warrants caution in interpreting these kind of tests.

In line with the results in adolescence, social interest was decreased in the 3 chamber task in adult male CH compared to standard housed animals, while social discrimination was not affected (Kentrop et al., 2018). These animals are continuously challenged and surrounded by many cage mates in their own home cage, which seems to make them less interested in social contact in a potentially less challenging context. Preliminary results now also point to impaired pro-social behavior in CH males in adulthood (see Kentrop et al., this issue), since CH males do not prefer the pro-social lever in a 2- choice task. We are currently testing their motivation to liberate a trapped cage mate.

The CH rodent model might provide an interesting starting point to observe the effects of a challenging and demanding environment in terms of peer presence. On the one hand such an environment appears to stimulate cognitive performance, as many beneficial effects of social enrichment have been observed in cognitive tasks (Gubert and Hannan, 2019); in agreement, we observed increased attention in the 5-choice task. But this seems to go together with a diminished social interest and decreased behavioral control, the experimental endpoints of our consortium.

### 4.2 Limited nesting and communal housing models in mice

An overview of all results obtained in our different mouse studies is given in Table 3. These studies were focused on maternal care and we have extensively measured this in the F1 (see results later on), but also in the wildtype F0 mothers, during the different early life conditions. We found that, compared to the standard housed condition, mothers in the ‘impoverished’ limited nesting and bedding condition showed more unpredictable behavior ; in the ‘enriched’ communal nesting condition there was more often a mother on the nest (Knop et al., 2020, [Knop et al., 2019] 2019). Also, in communal nests, there were more on/off nest transitions of the mothers, probably due to the fact of alternating nest presence of the two mothers. Considering the immediate consequences of the rearing conditions on the pups, we observed a lower bodyweight in pups at the end of the limited nesting condition, while communal housed pups were heavier (Knop et al., 2020, 2019). After weaning (results summarized in Table 3) limited nesting gives rise to lower bodyweight in adolescence and a delayed puberty onset compared to both standard and communal nesting conditions and irrespective of genetic background. To determine puberty onset, female mice were examined daily from postnatal day 24–36 on vaginal opening (Caligioni, 2009) and males were examined daily on preputial separation from postnatal day 27–32 (Korenbrot et al., 1977). The effects on puberty were partly mediated by the effects on bodyweight, an observation that is well known from both the human (Martos-Moreno et al., 2010) and animal literature (Caron et al., 2012), and likely represents a necessary minimum of bodily resources together with altered hormonal status to enter the reproductive phase. Bodyweight did not solely control puberty onset in our model, since the relation between the limited nesting and bedding condition and a delayed puberty onset was still present after controlling for bodyweight, pointing to other changes related to reproduction (Caron et al., 2012). Pubertal processes are linked to adolescent psychological development, most likely through hormonal effects (Berenbaum et al., 2015). There is evidence that pubertal development that is off-time in any way can increase the risk of psychopathology (Graber, 2013). Indeed deviations in pubertal timing are linked to anxiety, depression and social disorders, although there is a bias in studies towards girls compared to boys (Mendle et al., 2007; Mendle and Ferrero, 2012). Early life adversity has been shown to accelerate sexual maturation in both humans (Belsky et al., 2015; Mendle et al., 2011) and rodents (Cameron et al., 2008; Cowan and Richardson, 2019) and positive family relations were linked to a delay in puberty onset in humans (Graberc et al., 1995). We however, did not observe results in line with the life history theory in humans, predicting accelerated reproductive development in unsecure conditions (Belsky et al., 1991). This might be a species difference, but could also represent a limitation of the translational value of the animal model used. A model that would provide animals not only the balanced rodent chow to eat, but also a less healthy “fast-food like” diet, might be a better representation of the human situation (Roth and DiVall, 2016). Indeed, it was recently observed that animals adapt their food choice and intake based on early life experiences (Yam et al., 2017), which could subsequently influence puberty onset.

We observed limited effects of the early life conditions in our first study (with the MR^+/−^ mice) on the selected mouse social output measure, i.e. maternal care behavior of the F1 generation. Given the different trajectories of brain development involved in different behaviors, the effects of early life conditions likely depend on the timing of exposure (Casey et al., 2019; Fuhrmann et al., 2015; Hodes and Epperson, 2019; Rice and Barone, 2000; Semple et al., 2013). A recent study suggested that a later window of exposure to the limited nesting model could more clearly affect susceptibility to social defeat stress (Peña et al., 2019). We thus decided to extend our early life conditions to postnatal day 14 in the second study, which was carried out with DRD4^+/−^ mice. With this protocol, we indeed saw more pronounced effects of the early life conditions on maternal care in the F1 generation, irrespective of genetic background, specifically when comparing mice that were exposed to early life adversity with those experiencing early life enrichment. The female mice that were exposed to limited nesting when they were young themselves showed less nursing, typically less arched-back (active posture) nursing, less licking and grooming of their offspring, higher unpredictability on the nest and spent less time on the nest, compared to mothers that were exposed to a communal housing setting when they were young. These changes in maternal behavior do not literally copy the maternal behavior that the F1 dams received as pups, so there seems to be more than a pure intergenerational transmission of maternal care, which might also be apparent in other social behaviors.

Altogether our results strengthen the assumption that limited nesting and communal housing conditions are clearly different on the scale of early life experiences, and can in the future be used to model the effects of impoverished and enriched early life conditions respectively on other aspects of social behavior and social competence.

### 4.3 The CID rodent cohort: Relevance for human investigations

#### 4.3.1 Early life models

At the time that CID started, most rodent models for early life adversity focused on the prenatal or first postnatal weeks. These interventions are very powerful in provoking changes in the amygdala-hippocampus complex, causing altered anxiety and memory formation, as recently confirmed in an extensive meta-analysis (Bonapersona et al., 2019). Whether these models are also most suitable to study the outcome in terms of behavioral control and social behavior is less obvious. So far, our results in the animal cohort of CID indicate that interventions taking place at a later point of development are more effective regarding these behavioral endpoints. Thus, extending the period of limited nesting from postnatal day 9 to day 14 resulted in a stronger maternal care phenotype in female offspring. This is in line with other studies showing the impact of early life manipulations extending into the second postnatal week on social behavior (Kambali et al., 2019; Peña et al., 2019) and coincides with a developmental period of synaptic expansion, myelination and receptor changes (Semple et al., 2013). The moderate impact of the -in itself severe- early life conditions that we applied, also points to resilience in this early life period. Although we had hoped to model early life adversity leading to changes in social behavior, to be able to study the underlying neurobiological changes, the picture appears to be more complex: Development of (mal)adaptations in social behavior appear to take more than a very stressful event at one period in time. This is an important point to emphasize for future experiments. Today most currently used models still apply a single stressor in a confined period of life, while growing evidence supports that the ELA phenotype is strongest when combined with other negative experiences (Bonapersona et al., 2019; Daskalakis et al., 2013; Schmidt et al., 2011). Preferably, models of ELA should include (unpredictable) stressors on multiple time points in life and include (epi)genetic factors, to get a grip on what happens when either maladaptive social behavior develops or resilience is promoted (Hodes and Epperson, 2019; Kentner et al., 2019; Peña et al., 2019).

A particularly strong behavioral phenotype was observed in the complex housing model in rats. We originally used the complex housing condition as a possible condition that could rescue the early life effects. But it turned out that complex housing shapes a phenotype that had very marked effects on its own and does not interact -at least, for none of the experimental endpoints that we tested- with the maternal deprivation early life condition. We therefore learned to consider it as a condition on its own. This complex housing environment, which may model a condition of demanding peer-peer interactions from early adolescence onwards, is associated with increased attention in novel situations coupled to quick habituation, a higher degree of impulsiveness and reduced interest in peers, at least when studied under standard test conditions. It is tempting to compare this model with the complex environment in which many urban adolescents grow up nowadays, with an overflow of challenges, information and peer-peer interactions (Christakis et al., 2018). Of note, complex housing started at postnatal day 26 and lasted until testing in adulthood. Current experiments in the CID rodent cohort examine to what extent shorter stays in a complex environment, e.g. restricted to the adolescent period, are as effective in altering the behavioral phenotype in adulthood. Although the exact timing of early life interventions is still a matter of investigation, so far the animal studies suggest that adversity taking place very early in life -the equivalent of the perinatal period in humans- may have relatively limited consequences in the social domain.

Different from the human situation, most animal models of early life focus on maternal care, disregarding the role of the father. Although mice and rats are not necessarily biparental species, it is possible to involve the father; if the male has access to and sires only one female, he will engage in parental care (Wright and Brown, 2000). This paternal care has been shown to be dependent on communication with the mother (Liu et al., 2013). Help of the father could counteract negative effects of harsh conditions, and even be used as an intervention in rodent models. Also, paternal stress phenotypes can be transmitted to the next generation via epigenetic mechanisms (Cunningham et al., 2019; van Steenwyk et al., 2018) and influence vulnerabilty or resilience to stress in the offspring.

Another obvious difference between the rodent and human situation concerns the number of the offspring. Rodent litters involve multiple pups, which is very different from the human situation. Indeed pups coming from smaller litters generally grow and develop faster compared to pups from bigger litters, since the dam has a limited amount of resources, both intrauterine and in postnatal milk supply (Galler and Turkewitz, 1975; Wehmer and Jen, 1978); hence the importance of culling to obtain litters of comparable size. Here we obviously miss out on face validity. All of these aspects add to the need of extreme care when we translate our findings in rodents to the human situation or vice versa. Interestingly however, differences in parenting towards different ‘children’ within a family is also observed within rat litters (Claessens et al., 2011; van Hasselt et al., 2012v).

#### 4.3.2 Assessing social competence

As is evident from the systematic review, the repertoire of social tasks in rodents was rather limited at the start of the consortium. Especially in the realm of pro-social behavior, relevant tasks were scarce. We therefore redesigned existing tasks into new paradigms, of which one (the fully automated, operant pro-social two-choice task) is described in more detail elsewhere in this special issue. In this task, which is a decision-making task, we observed a consistent 60 % preference for the pro-social choice in standard-housed rats, which is rather low but not very different from percentages observed in other studies with a similar set-up (Hernandez-Lallement et al., 2015). Active training of the partner rat to guide behavior of the test rat gave higher preference levels (Márquez et al., 2015) and might be something to integrate in future experiments. Still, we of course only capture in our rodent model a specific component of pro-social decision making, which might represent a building block of the more complex behavior we observe in humans (Juavinett et al., 2018).

In the selection of behavioral tasks, we aimed to align the animal experiments as much as possible with the human investigations. It should be realized though, that in the operant tasks (5-CSRTT, 2 -choice reward task and pre-training in the liberation task), animals are mildly food-deprived to increase learning of lever pressing for a sucrose reward. This is something which is obviously not done in human studies. Next to this, many of the tasks we have performed (like the operant tasks) require daily sessions, starting with habituation and training to gain stable performance, which is then followed by the testing phase. Each of these tasks takes about 2.5 months of testing for the animal. This is quite a long period, and experiments like these are intensive, not only for the animal, but also for the experimenter. However, a big advantage of measuring continuous performance and behavior over several weeks is that the thus established characteristics are less likely to be influenced by arbitrary daily factors that might influence shorter versions of the tests.

Finally, interpreting rodent behavior is inherently difficult, because one has to infer a meaning from what can be seen by the experimenter. For instance, opening the door for a trapped rat can point to empathy in the test rat but can also be driven by the prospect of social reward. It requires thorough validation of the task -e.g. by also allowing an option in which the trapped rat is freed but without the benefit of social contact for the test rat- to narrow down the interpretations of the behavior exhibited.

#### 4.3.3 Exploiting the full potential of the rodent cohort

Despite all of these limitations, we consider the presence of a rodent cohort in parallel with the human cohorts in CID as an added value. We will illustrate this with three examples.

The first example shows how animal studies can give rise to new hypotheses that can be tested in human cohorts. In our mouse studies we observed that a high degree of unpredictability in maternal care is linked to a later puberty onset, an effect that is mediated by bodyweight (Knop et al., 2019). This question is currently being followed up in one of the CID human cohorts. Even if the mouse and human data don’t congrue, investigation of the differences between the conditions can lead to valuable insights.

In the second example, studies in humans guide the animal experiments. Human studies have supplied evidence for differential susceptibility, i.e. the phenomenon that a certain genetic background makes individuals more (or less) susceptible to the environment, for better or for worse (Belsky and van IJzendoorn, 2017). The human dopamine receptor subtype 4 (DRD4) is supposed to be one of the genes important for differential susceptibility (Bakermans-Kranenburg et al., 2008; Windhorst et al., 2015). However, the genetic and environmental variability in humans is notorious. We therefore used a mouse study with tight control over genetic background (particularly the DRD4 gene) and environment to confirm the existence of differential susceptibility, in this case with regard to maternal care in the female offspring (Knop et al., 2020).

The advantage of reproducing human findings in animal models is that one can follow the latter up with detailed neurobiological observations to better understand the underlying mechanism, up to the level of single cells. This is currently not feasible in the human brain, despite advances in neuroimaging techniques. This value is illustrated by the final example. Thus, we demonstrated that 24 h MD on postnatal day 3 predominantly affects (spatial) memory formation rather than reward-based decision making (Loi et al., 2017); and that this can be prevented by treatment with an anti- glucocorticoid between postnatal days 26 and 28. At the single cell level, this was mirrored by changes in glutamate transmission that could be alleviated by the 3 days’ antiglucocorticoid treatment, particularly in those areas involved in spatial memory formation. This deeper level of analysis suggests that glutamate transmission might be one of the systems targeted by early life adversity, subsequently leading to altered behavior. Clearly, the advantages of such mechanistic experiments can be much better exploited in future studies in CID, thereby nicely complementing the advantages of studies in human cohorts. The interknitted design of the consortium, in which animal and human paradigms were aligned as much as possible, will allow us to fully exploit the translational potential once more data will become available.

## Declaration of Competing Interest

The authors declare that they have no known competing financial interests or personal relationships that could have appeared to influence the work reported in this paper.
