# Supplementary material for: The relevance of a rodent cohort in the Consortium on Individual Development
Source: Dev Cogn Neurosci. 2020 Aug 27;45:100846. doi: 10.1016/j.dcn.2020.100846 (PMC7509002; doi:10.1016/j.dcn.2020.100846)
Supplement: Supplementary file 1 [file mmc1.docx]

Supplementary methods: Search string

Search string for the systematic literature search in PubMed and WebOfScience (described in section 3.1). Since we were updating a previous search (Bonapersona et al., 2019), a date filter was applied (publications after the 1^st^ of February 2017). This allowed for an overlap of a few months between the previous search and the date filter of the current search.

**Pubmed**

(

"early life stress"[tiab] OR "ELS"[tiab] OR "early life adversity"[tiab] OR "early life adversities"[tiab] OR "early life adversity*" OR "early stress"[tiab] OR "neonatal stress"[tiab] OR "postnatal stress"[tiab] OR "perinatal stress"[tiab] OR "neonatally stressed"[tiab] OR "early adverse experience"[tiab] OR "perinatally stressed"[tiab] OR "early adverse experiences"[tiab] OR "postnatal manipulation"[tiab] OR "postnatal manipulations"[tiab] OR "perinatal manipulation"[tiab] OR "perinatal manipulations"[tiab] OR "limited bedding"[tiab] OR "limited nesting"[tiab] OR "limited material"[tiab] OR "licking and grooming"[tiab] OR "licking-grooming"[tiab] OR "licking/grooming"[tiab]

) AND (

"murine"[tiab] OR "rodentia"[tiab] OR "rodent"[Tiab] OR "rodents"[Tiab] OR "rodentia"[tiab] OR mus[Tiab] OR murinae[Tiab] OR muridae[Tiab] OR "mice"[MeSH Terms] OR "mice"[tiab] OR "mouse"[tiab] OR "rats"[MeSH Terms] OR "rat"[tiab] OR "rats"[tiab]

) AND (

"Behavior, Animal"[Mesh] OR "behaviour"[tiab] OR "behavior"[tiab] OR "behaviours"[tiab] OR "behaviors"[tiab] OR "behav*"[tiab] OR "behavioural test"[tiab] OR "behavioural tests"[tiab] OR "behavioral test"[tiab] OR "behavioral tests"[tiab] OR "test, behavioral"[tiab] OR "test, behavioural"[tiab] OR "tests, behavioral"[tiab] OR "tests,behavioural"[tiab] OR "anxiety"[tiab] OR "fear"[tiab] OR "anxiety/fear"[tiab] OR "anxiety-fear"[tiab] OR "emotional learning"[tiab] OR "non-stressful learning"[tiab] OR "non stressful learning"[tiab] OR "social behaviour"[tiab] OR "social behavior"[tiab] OR "sexual behaviour"[tiab] OR "sexual behavior"[tiab] OR "anxiety"[tiab] OR "fear"[tiab] OR "anxiety/fear"[tiab] OR "anxiety-fear"[tiab] OR "emotional learning"[tiab] OR "non-stressful learning"[tiab] OR "non stressful learning"[tiab] OR "social behaviour"[tiab] OR "social behavior"[tiab] OR "sexual behaviour"[tiab] OR "sexual behavior"[tiab] OR "radial arm"[tiab] OR "T maze"[tiab] OR "Ymaze"[tiab] OR "what where which task"[tiab] OR "what-where-which task"[tiab] OR "object in location"[tiab] OR "object in context"[tiab] OR "object recognition"[tiab] OR "object discrimination"[tiab] OR "barnes maze"[tiab] OR "holeboard"[tiab] OR "circular maze"[tiab] OR "Morris water maze"[tiab] OR "spontaneous alteration task"[tiab] OR "maze learning"[tiab] OR "active avoidance"[tiab] OR "spring test"[tiab] OR "inhibitory avoidance"[tiab] OR "passive avoidance"[tiab] OR "defensive withdrawal"[tiab] OR "fear conditioning"[tiab] OR "cat box"[tiab] OR "elevated plus maze"[tiab] OR "EPM"[tiab] OR "cross maze"[tiab] OR "open field"[tiab] OR "concentric square field test"[tiab] OR "place preference"[tiab] OR "place avoidance"[tiab] OR "light/dark test"[tiab] OR "light dark test"[tiab] OR "light-dark test"[tiab] OR "light/dark box"[tiab] OR "light dark box"[tiab] OR "light-dark box"[tiab] OR "object exploration"[tiab] OR "square field test"[tiab] OR "shuttle box"[tiab] OR "social interaction"[tiab] OR "three chambers"[tiab] OR "3 chambers"[tiab] OR "three chamber"[tiab] OR "3 chamber"[tiab] OR "1 chamber"[tiab] OR "one chamber"[tiab] OR "emotional witness stress"[tiab] OR "social play"[tiab] OR "social approach test"[tiab] OR "social encounter test"[tiab] OR "social interaction test"[tiab] OR "social preference test"[tiab] OR "social learning"[tiab] OR "social preference"[tiab] OR "social hierarchy"[tiab] OR "dominance"[tiab] OR "tube test"[tiab] OR "resident test" [tiab] OR "intruder test"[tiab] OR "resident intruder test"[tiab] OR "resident/intruder test"[tiab] OR "resident-intruder test"[tiab] OR "competitive behaviour"[tiab] OR "competitive behaviour"[tiab] OR "play fighting behaviour"[tiab] OR "play fighting behaviour"[tiab] OR "play-fighting behaviour"[tiab] OR "play-fighting behavior"[tiab] OR "play/fighting behaviour"[tiab] OR "play/fighting behavior"[tiab]

)

**Web of Science**

(

"early life stress" OR "ELS" OR "early life adversity" OR "early life adversities" OR "early life adversity*" OR "early stress" OR "neonatal stress" OR "postnatal stress" OR "perinatal stress" OR "neonatally stressed" OR "early adverse experience" OR "perinatally stressed" OR "early adverse experiences" OR "postnatal manipulation" OR "postnatal manipulations" OR "perinatal manipulation" OR "perinatal manipulations" OR "limited bedding" OR "limited nesting" OR "limited material" OR "licking and grooming" OR "licking-grooming" OR "licking/grooming"

) AND (

"murine" OR "rodentia" OR "rodent" OR "rodents" OR "rodentia" OR mus OR murinae OR muridae OR "mice" OR "mouse" OR "rat" OR "rats"

) AND (

"behaviour" OR "behavior" OR "behaviours" OR "behaviors" OR "behav*" OR "behavioural test" OR "behavioural tests" OR "behavioral test" OR "behavioral tests" OR "test, behavioral" OR "test, behavioural" OR "tests, behavioral" OR "tests,behavioural" OR "anxiety" OR "fear" OR "anxiety/fear" OR "anxiety-fear" OR "emotional learning" OR "non-stressful learning" OR "non stressful learning" OR "social behaviour" OR "social behavior" OR "sexual behaviour" OR "sexual behavior" OR "anxiety" OR "fear" OR "anxiety/fear" OR "anxiety-fear" OR "emotional learning" OR "non-stressful learning" OR "non stressful learning" OR "social behaviour" OR "social behavior" OR "sexual behaviour" OR "sexual behavior" OR "radial arm" OR "T maze" OR "Ymaze" OR "what where which task" OR "what-where-which task" OR "object in location" OR "object in context" OR "object recognition" OR "object discrimination" OR "barnes maze" OR "holeboard" OR "circular maze" OR "Morris water maze" OR "spontaneous alteration task" OR "maze learning" OR "active avoidance" OR "spring test" OR "inhibitory avoidance" OR "passive avoidance" OR "defensive withdrawal" OR "fear conditioning" OR "cat box" OR "elevated plus maze" OR "EPM" OR "cross maze" OR "open field" OR "concentric square field test" OR "place preference" OR "place avoidance" OR "light/dark test" OR "light dark test" OR "light-dark test" OR "light/dark box" OR "light dark box" OR "light-dark box" OR "object exploration" OR "square field test" OR "shuttle box" OR "social interaction" OR "three chambers" OR "3 chambers" OR "three chamber" OR "3 chamber" OR "1 chamber" OR "one chamber" OR "emotional witness stress" OR "social play" OR "social approach test" OR "social encounter test" OR "social interaction test" OR "social preference test" OR "social learning" OR "social preference" OR "social hierarchy" OR "dominance" OR "tube test" OR "resident test" OR "intruder test" OR "resident intruder test" OR "resident/intruder test" OR "resident-intruder test" OR "competitive behaviour" OR "competitive behaviour" OR "play fighting behaviour" OR "play fighting behaviour" OR "play-fighting behaviour" OR "play-fighting behavior" OR "play/fighting behaviour" OR "play/fighting behavior"

)
